# Exploring the Reconfigurable Memory Effect in Electroforming-Free YMnO_3_-Based Resistive Switches: Towards a Tunable Frequency Response

**DOI:** 10.3390/ma17112748

**Published:** 2024-06-05

**Authors:** Xianyue Zhao, Nan Du, Jan Dellith, Marco Diegel, Uwe Hübner, Bernhard Wicht, Heidemarie Schmidt

**Affiliations:** 1 Institute for Solid State Physics, Friedrich Schiller University Jena, 07743 Jena, Germany; xianyue.zhao@uni-jena.de (X.Z.); nan.du@uni-jena.de (N.D.); 2 Leibniz Institute of Photonic Technology (IPHT), 07745 Jena, Germany; jan.dellith@leibniz-ipht.de (J.D.); marco.diegel@leibniz-ipht.de (M.D.); uwe.huebner@leibniz-ipht.de (U.H.); 3 Institute of Microelectronic Systems and Laboratory of Nano and Quantum Engineering, Leibniz University Hannover, 30167 Hannover, Germany; bernhard.wicht@ims.uni-hannover.de

**Keywords:** YMnO_3_-based resistive switches, electroforming-free resistive switching, reconfigurable memory effect, impedance circuit, tunable frequency response, ultra-high reconfigurability, charged domain walls, vortices, conducting filaments

## Abstract

Memristors, since their inception, have demonstrated remarkable characteristics, notably the exceptional reconfigurability of their memory. This study delves into electroforming-free ${\mathrm{YMnO}}_{3}$ (YMO)-based resistive switches, emphasizing the reconfigurable memory effect in multiferroic YMO thin films with metallically conducting electrodes and their pivotal role in achieving adaptable frequency responses in impedance circuits consisting of reconfigurable YMO-based resistive switches and no reconfigurable passive elements, e.g., inductors and capacitors. The multiferroic YMO possesses a network of charged domain walls which can be reconfigured by a time-dependent voltage applied between the metallically conducting electrodes. Through experimental demonstrations, this study scrutinizes the impedance response not only for individual switch devices but also for impedance circuitry based on YMO resistive switches in both low- and high-resistance states, interfacing with capacitors and inductors in parallel and series configurations. Scrutinized Nyquist plots visually capture the intricate dynamics of impedance circuitry, revealing the potential of electroforming-free YMO resistive switches in finely tuning frequency responses within impedance circuits. This adaptability, rooted in the unique properties of YMO, signifies a paradigm shift heralding the advent of advanced and flexible electronic technologies.

## 1. Introduction

The emergence of memristive switches in 2008 [1] marked the inception of a revolutionary era in electronic components distinguished by exceptional properties. These distinctive devices, predicted in 1971 by Leon Chua [2], have found extensive applications in in-memory computing, encompassing brain-inspired, and logic in-memory computing. Beyond their proficiency in memory-related domains, the favorable electrical characteristics of memristors have propelled their integration into diverse electronic circuits, underscoring their significance in the dynamic landscape of electronic devices.

Numerous research endeavors have explored the utilization of memristors across a spectrum of electronic circuits [3,4,5], including amplifiers, filters, oscillators, and more. Noteworthy investigations have focused on the transmission characteristics of non-inverting operational amplifier circuits based on memristors with linear dopant drift [6]. Studies have expanded to encompass the analysis of monotone increasing and piecewise-linear nonlinear memristor models in both inverting and non-inverting operational amplifier circuits [7,8]. In the realm of signal processing, memristors have been leveraged to enhance ultra-wideband systems [9,10,11], thereby reducing signal processing power. Additionally, they have been applied in fine-resolution programmable resistors, programmable gain amplifiers, and differential amplifiers [12,13,14]. The impact of memristors extends to their role in filter circuits, integrating with capacitors in first-order low-pass filters for sensing resistive memory and showcasing experimental demonstrations of adaptive band-pass filters within LC contours [15].

Despite the extensive exploration of memristor applications in crossbar arrays through simulation-based studies, a notable gap remains in experimental validations and in the discovery of electroforming-free memristors. Most prominent memristors with large memory windows, e.g., TiO_2_, HfO_2_, and TaO_2_ [16,17,18], still need an electroforming step which hinders upscaling of memristor crossbar arrays. This study addresses this gap by focusing on electroforming-free resistive switches based on ${\mathrm{YMnO}}_{3}$ (YMO). The multiferroic YMO has charged domain walls and vortices, which can be considered conducting filaments. The vortex density is defined as the number of vortex points in a cutting plane through the ferroelectric manganites and can be measured using, for example, piezo force microscopy measurements [19]. Electroforming-free resistive switching and the existence of vortices in polycrystalline hexagonal YMO thin films strongly hint towards a relation between the conductive filaments and the vortices rather than towards a relation between conductive filaments and defects [20].

These electroforming-free switches present a compelling avenue for exploration, featuring ultra-high reconfigurability. Building upon our prior study, which successfully detected polarization charges and identified polarization directions (±Pz) within polycrystalline YMO thin films using scanning electron microscopy (SEM) in secondary electron emission mode [21], this work not only delves into the exploration of these resistive switches but also develops an equivalent model for their behavior. The equivalent model exploits the fact that the vortex density in hexagonal YMO can be controlled by electric field ramps [20]. Importantly, we bridge the gap between theory and practice by experimentally demonstrating their potential in tuning frequency responses within impedance circuits. This work serves as a testament to the practical analog applications of YMO-based resistive switches, ushering in new possibilities for their integration into cutting-edge electronic systems.

## 2. Reconfigurability of Electroforming-Free Resistive Switches Based on Manganite Thin Film

In the realm of information technologies, which continuously consume an escalating portion of global energy resources, there is an imperative demand for multifunctional materials capable of mitigating the energy demands of microelectronic devices [22]. Memristors, categorized as multiferroic materials [2], with notable examples such as BiFeO_3_ (BFO) [23] and YMO [24], showcase the ability to dynamically reconfigure resistance states, transitioning between a high-resistance state (HRS) and low-resistance state (LRS) in response to an appropriate voltage bias or current stimulus. Typically, an electroforming process [16] is necessary to initiate the reconfigurable behavior and define the desired electrical properties by applying one or multiple large biases across the device. If no large bias is applied to initiate the process, this type of resistive switching is termed electroforming-free resistive switching. Electroforming-free resistive switching holds significant promise for implementing memory reconfigurability into upscaled crossbar arrays. To unravel the physical mechanisms underlying resistive switching in the electroforming-free, bipolar, analog-resistive switch BFO, our analysis focused on its impedance (*Z*), a parameter that encompasses both magnitude and phase (Z=Re{Z}+j·Im{Z}). In contrast to resistance, which solely considers magnitude (Re{Z}=R), impedance provides a comprehensive view. Our scrutiny of BFO memristors in both HRS and LRS [25] confirmed that the capacitance of the depletion layers at the top and bottom electrodes of the BFO memristor changes in tandem with the barrier height of the electrodes. It is substantial in HRS and diminished in LRS, shedding light on the underlying physical mechanism [26].

Unlike the electroforming-free, bipolar, analog switching BFO memristor [23], the YMO memristor is electroforming-free, unipolar, and exhibits abrupt switching behavior [27]. Our exploration of the transport properties of YMO thin films in HRS and LRS, utilizing temperature-dependent resistance measurements, reveals that hopping transport dominates in HRS, while metallic transport prevails in LRS [20].

**Figure 1:**
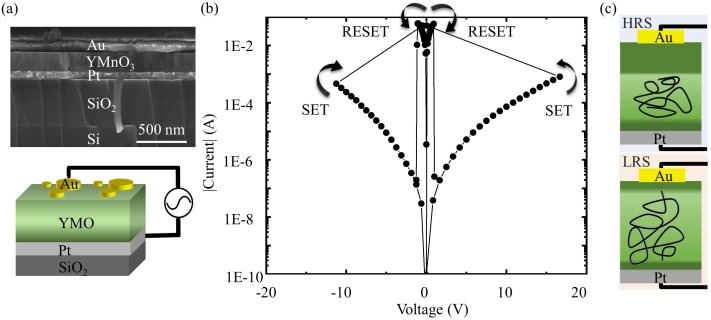
Structural and transport properties of an electroforming-free YMO-based resistive switch. (**a**) SEM view of the YMO-based resistive switch with a YMO layer thickness of 230 nm (length scale bar: 500 nm). Lower part of (**a**), schematic illustration of room temperature DC electrical measurement setup for YMO-based resistive switch connected in series with a DC source meter. (**b**) I–V characteristics for SET and RESET processes of resistive switches in both positive and negative bias ranges. (**c**) Illustration of the redistribution of vortices in the region close to the Au top electrode and Pt bottom electrode in a YMO resistive switch in LRS and HRS.

In our study, the YMO resistive switch is synthesized through pulsed laser deposition (PLD). A YMO film is deposited on a Pt/SiO_2_/Si substrate. As analyzed by SEM, the thicknesses of Au, YMO, Pt, and SiO_2_ layers amount to 150 nm, 230 nm, 85 nm, and 560 nm, respectively. The PLD-fabricated YMO thin film used in this work exhibits a polycrystalline structure. Laser deposition parameters, including laser energy (*E*), repetition rate (*f*), substrate temperature ($T_{s}$), oxygen partial pressure ($pO_{2}$), and the total number of laser pulses ($N_{r}$), are as follows: E=2.5 J/cm^3^, f=3 Hz, $T_{s}=800$ °C, $pO_{2}=0.18$ mbar, and $N_{r}=6000$, respectively. The substrates, consisting of a Pt (85 nm)/SiO_2_ (560 nm)/Si structure, are procured from Hefei Kejing Materials (China). The SiO_2_ layer, 560 nm in thickness, is grown via oxidation at 700 °C using Chemical Vapor Deposition (CVD). Subsequently, the Pt layer is deposited by direct current (DC) sputtering, forming an 85 nm-thick Pt layer on the SiO_2_/Si structure. The Pt layer serves as the bottom electrode, whereas the circular top electrodes, fabricated from Au with a diameter of 550 µm, are precisely formed using DC magnetron sputtering. As depicted in the scanning electron microscopy (SEM) image in Figure 1a, the morphology of the polycrystalline YMO layers reveals a dense structure, exhibiting uniform and coherent interfaces with both the Pt bottom electrode and the Au top electrode. The unipolar switching properties of the current–voltage (I–V) characteristics are recorded by the Keithley 4200A-SCS parameter analyzer (Keithley, Cleveland, OH, USA) in the SET and RESET states of YMO resistive switches, which are illustrated in Figure 1b. Upon the application of a positive writing voltage (0 → +25 V) with a compliance current, a sudden switching event occurs at the SET voltage (+V*_SET_*). Subsequently, a sweeping voltage in the range of (0 → +5 V) with a maximum current flow of approximately 100 mA leads to an abrupt decrease in current. For negative voltage sweeps of (0 →−25 V) and (0 →−5 V), analogous SET and RESET processes are observed, respectively. This unipolar behavior in the I–V characteristics underscores the reliable and repeatable switching performance of YMO resistive switches.

**Figure 2:**
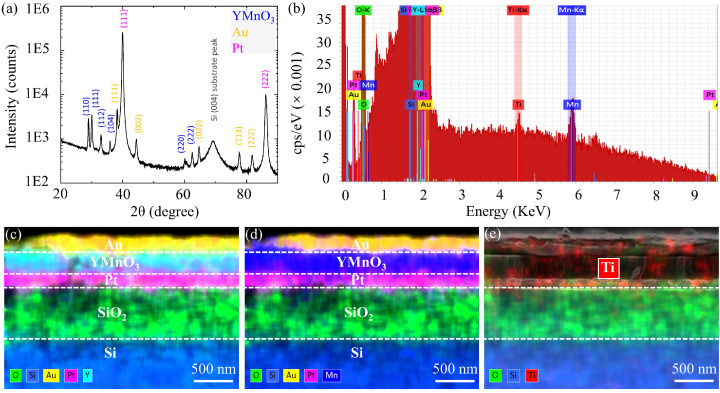
(**a**) XRD patterns of the YMO memristor, showing YMO (in blue), Au (in gold), and Pt (in pink). The Si (004) substrate peak is significantly suppressed due to the film stack thickness. (**b**) EDX spectrum of YMO memristor (Ti-KL3 radiation @ 4.5 keV). (**c**,**d**) Elemental mapping analysis of YMO memristor. (**e**) Elemental mapping shows Ti dispersed across the Au, YMO, and Pt layers, with partial distribution in the SiO_2_ layer, indicating contamination during the PLD process at 800 °C.

Figure 2a presents X-ray diffraction analysis (XRD) patterns (2θ-ω-scan) of the YMO memristor, which confirms the formation of a polycrystalline YMO phase with a hexagonal structure (space group P63cm). The diffraction peaks at 2θ = 28.9°, 30.0°, 33.1°, 60.1°, and 62.5° correspond to the (110), (111), (112), (220), and (222) planes of YMO, respectively. Ferroelectricity induced in hexagonal YMO is due to electrostatic and geometrical effects. It emerges in two transition steps [28,29]: at the ferroelectric Curie temperature, T_*C*_ = 1270 K, the centrosymmetric crystal structure P63/mmc is lowered to P63cm by the condensation of the K3 nonplanar mode [30,31], and a secondary mode provides ferroelectric polarization by corrugation of Y^3+^ planes with spontaneous polarization of 5.6 $\mu C\cdot {\mathrm{cm}}^{-2}$ [31,32]. Figure 2b presents the energy-dispersive X-ray spectrometry (EDX) spectrum (Ti-KL3 radiation @ 4.5 keV), and Figure 2c–e displays the elemental mapping analysis, offering valuable insights into the film of the YMO memristor. In Figure 2b, the EDX spectrum illustrates the distribution of different elements within the stack, with each color representing a specific element. The spectrum is recorded at an excitation energy of $E_{0}=10$ keV. Additionally, the ordinate scale is magnified to enhance the visibility of weak peaks such as Ti-K_α_ or Mn-K_α_. Energy windows are defined to facilitate element separation for EDX mappings, which are displayed underneath. Figure 2c,d displays the corresponding elemental mapping analysis, revealing a uniform distribution of Y, Mn, and O elements across the memristor’s cross-section. False colors are assigned to all detected elements (according to the energy window colors in the spectrum) to illustrate the location and spatial distribution of the film elements. This outcome aligns well with XRD analysis and the findings of our previous research [33]. Figure 2e shows the elemental mapping, demonstrating a small amount of Ti dispersed across the Au, YMO, and Pt layers, with partial distribution into the SiO_2_ layer. This indicates YMO fabrication contamination during the PLD deposition process at 800 °C.

It is important to highlight the electroforming-free resistive switching characteristics of YMO memristors in contrast to other memristive devices exhibiting abrupt switching by several orders of magnitude [16,17,18]. Unlike many of its counterparts requiring an electroforming process for repeatable SET and RESET switching behaviors, YMO stands out by demonstrating switching behaviors without the necessity of an electroforming step. This unique attribute underscores the exceptional nature of YMO, enhancing the yield and reliability of YMO memristors in electronic applications, thereby contributing to their distinctiveness in the realm of resistive switching devices. To elucidate this distinctive feature, we propose that manipulating vortex density within YMO can be achieved by applying a specific bias-time profile between the top and bottom electrodes of the YMO-based resistive switch, as illustrated in Figure 1c. In its initial state, this switch resides in HRS, and following the application of an electrical stimulus, it undergoes a transition to LRS. This controlled transition between HRS and LRS is integral to the unique functionality of the YMO-based resistive switch, allowing for the manipulation of vortex density in response to the applied bias-time profile. In this context, we propose that the vortex density within YMO can be effectively decreased by applying an alternative bias-time profile, resulting in a transition from LRS to HRS, as illustrated in Figure 1c. This ability to manipulate vortex density bidirectionally through distinct bias-time profiles enhances the versatility of the YMO-based resistive switch. Support for our proposal is found in relevant studies in the literature. As an example, Meier et al. [34] conducted research on how the vortex density in YMO is influenced by the cooling rate when the material undergoes cooling below its ferroelectric ordering temperature. Additionally, Skjærvø et al. [35] explored the unusual continuous structural disorder in multiferroic YMO, uncovering anomalies in both local and average structural analyses as the material approaches its Curie temperature. These findings indicate increased fluctuations in the order-parameter angle, shedding light on the complex dynamics influencing the vortex density within YMO.

Concerning the electroforming-free YMO-based resistive switch’s reconfiguration from LRS to HRS, we propose the model illustrated in Figure 1c. In the LRS state, the carriers transporting currents are localized within vortices and topologically protected. These carriers can drift in the electric field generated by the applied bias *U*, gaining electrical energy e·U, which can be related to thermal energy $k_{B}\cdot T$, where $k_{B}$ is the Boltzmann constant, and *T* is the absolute temperature in Kelvin. If the temperature exceeds the ordering temperature, the ferroelectric ordering and the previously topologically protected carrier transport within vortices are no longer preserved. Due to the unconventional continuous structural disorder observed in YMO at the ferroelectric ordering temperature [35], enhanced interaction between carriers and the lattice is expected. Consequently, the carriers forming the vortices will cool down. For RESET (LRS → HRS) (Figure 1b), a cooling rate above the ordering temperature is anticipated, leading to a reduction in vortex density as ferroelectric ordering is re-established.

In contrast, for the reconfiguration of the YMO-based resistive switch from HRS to LRS, we propose the following model. When YMO is in HRS, hopping transport dominates within the YMO lattice, rendering transport within the topologically protected vortices negligible. Drawing upon a model by Lin et al. [36], who investigated the winding number of vortices in manganites using a network consisting of closed-loop and open-loop vortices and found a high probability for the formation of open-loop vortices with a winding number of 1, we suggest the following model. There are no open-loop vortices in contact with the top and bottom electrodes in the YMO-based resistive switch in HRS. Therefore, when ramping the bias, e.g., from 0 V to ±25 V (Figure 1b), an electric field *E* larger than |E|>2000 kV/cm is formed between the top and bottom electrodes. This field causes significant elastic deformation of the polycrystalline YMO material, known for its large magnetoelastic coupling constant [37]. Under these conditions, the network of open-loop and closed-loop vortices undergoes a transformation until the first open-loop vortex in contact with the top and bottom electrodes is formed and shunts the YMO-based resistive switch. After shunting, the bias cannot be ramped further. For SET (HRS → LRS) (Figure 1b), we anticipate a restructuring of the network of vortices until the first vortex shunts the top and bottom electrode of the YMO-based resistive switch.

Before incorporating the YMO-based resistive switch into more intricate impedance circuitry with capacitors and inductors to tune its frequency response, a comprehensive analysis of the impedance response in the frequency domain is conducted. This analysis, crucial for optimizing performance and effective integration into frequency-tunable circuitry, is performed after reconfiguring the switches into both the LRS and HRS.

**Figure 3:**
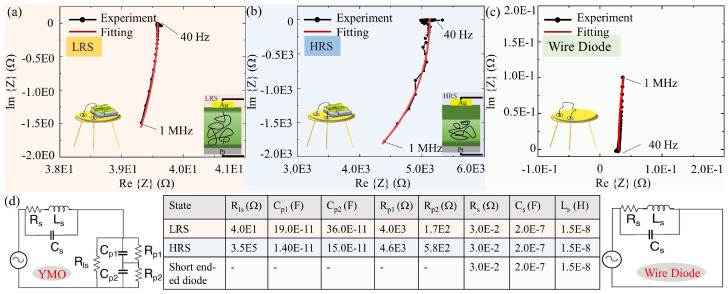
Measured and modeled impedance of the YMO-based resistive switch wire-bonded on a diode socket in (**a**) LRS and in (**b**) HRS. (**c**) Measured and modeled impedance of the short ended diode socket. Insets show (**a**–**c**) diode for impedance measurements and (**a**,**b**) the distribution of vortices. The voltage amplitude and frequency range of the AC signal for the resistive measurements are 50 mV and 40 Hz–1 MHz, respectively. (**d**) Equivalent circuits and modeling parameters of a YMO-based resistive switch wire-bonded on a diode socket and of a short ended diode socket.

The illustrated Figure 3a,b depicts the measured and modeled real part (Re{Z}) as well as the imaginary part (Im{Z}) of the impedance response of a YMO-based resistive switch on a diode socket. Figure 3c shows the measured and modeled impedance of the short-ended diode socket. These impedance responses are obtained by applying a 50 mV alternating current (AC) small-signal frequency sweep spanning from 40 to 1 MHz. A notable distinction between LRS and HRS is observed, particularly concerning the intersection point of the impedance data with the real axis (Re{Z}), indicating a substantial difference in resistivity between the two states. Furthermore, the imaginary part of the impedance response (Im{Z}) in both LRS and HRS extends into the negative imaginary quadrant with increasing testing frequency, suggesting the presence of a capacitive component in both states, contributing to the overall impedance characteristics of the YMO resistive switch.

An equivalent-circuit model has been developed to provide a unified representation for both the LRS and HRS of the YMO resistive switch, as shown in Figure 3d. This model, designed for a thorough exploration of the impedance response, consists of a resistance ($R_{\mathrm{ls}}$) operating in parallel with two sets of parallel-connected capacitance//resistance circuits ($C_{p1}$//$R_{p1}$ and $C_{p2}$//$R_{p2}$).

In LRS, the current is carried by the vortices, and the resistance $R_{\mathrm{ls}}$ can be derived from the intersection point of the impedance data with the real axis, which amounts to 40 Ω, characterizing the AC transport through both the closed- and open-loop vortex. In HRS, the combination of series-connected $R_{p1}$ and $R_{p2}$ representing the intersection point of the impedance data with the real axis, i.e., 5.2 × 10^3^ Ω, underscores an impressive 130 times difference in resistive values between the two states. This substantial contrast establishes a foundation for the design of frequency-tunable circuits. The two sets of parallel-connected capacitance/resistance circuits elucidate the AC transport within the surrounding YMO matrix, sharing analogous values for $R_{p1}$ and $R_{p2}$ in both LRS and HRS. It is significant to note that the capacitance $C_{p1}$ and $C_{p2}$ within the LRS are 19.0 × 10^−11^ F and 36 × 10^−11^ F, respectively, markedly surpassing those within the HRS, which are measured at 1.4 × 10^−11^ F and 15 × 10^−11^ F. This substantial discrepancy in capacitance between LRS and HRS aligns with findings from impedance responses in similar filamentary memristor devices [38,39], where the depletion layer extension in the YMO thin film below the metallic conducting electrodes is significantly reduced in the LRS state in comparison to the HRS state.

Furthermore, we conducted impedance testing on the same diode socket short-ended with gold wiring between two pins and developed a model to analyze the impedance response. The model for the short-ended diode socket incorporates a series resistance ($R_{s}$), parasitic capacitance ($C_{s}$), and parasitic inductance ($L_{s}$). Exclusively focusing on the impedance response from the short ended diode socket, we effectively capture the influence of electrodes on the real and imaginary components of the complex impedance, denoted as $R_{s}$ = 3.0 × 10^−2^ Ω, $C_{s}$ = 2.0 × 10^−7^ F, and $L_{s}$ = 1.5 × 10^−8^ H. These values are utilized in the impedance modeling of the YMO resistive switch in both LRS and HRS. The key parameters derived from the modeling process, encompassing parallel capacitance values ($C_{p}$), parallel resistance ($R_{p}$), series inductance ($L_{s}$), and series resistance ($R_{s}$) for YMO resistive switches in both the LRS and HRS, as per the proposed equivalent circuits, are succinctly summarized in Figure 3d.

## 3. Reconfigurable Impedance Circuitry

In the following sections, we seamlessly integrate YMO resistive switches with commercially available capacitors and inductors, creating memristor–capacitor (M-C) and memristor–inductor (M-L) circuits, respectively. This integration emphasizes the remarkable reconfigurability in the frequency response achieved when the YMO resistive switch operates in either LRS or HRS within the impedance circuits. The demonstrated M–C or M–L circuits, characterized by tunable frequency responses, can be conceptualized as inductor–resistor (L-R) or capacitor–resistor (C-R) circuits with a reconfigurable resistor. These configurations open up possibilities for filter applications with adaptable cut-off frequencies.

**Figure 4:**
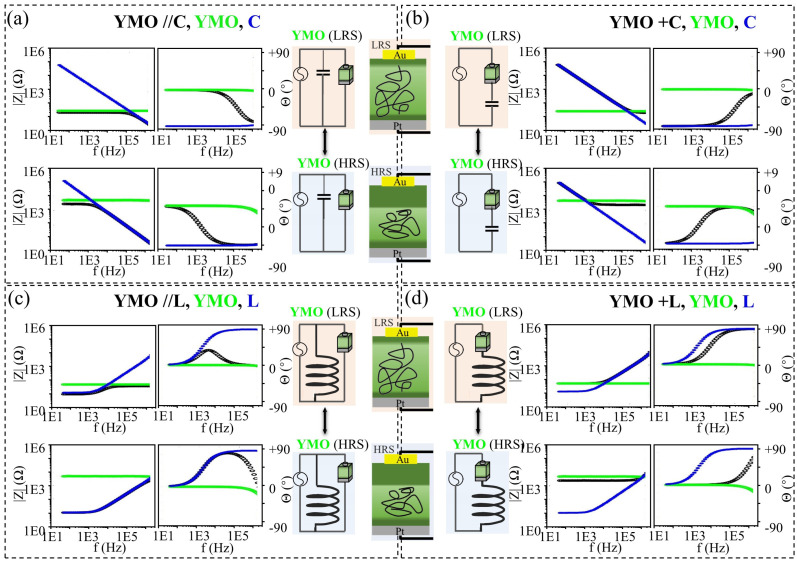
Measured magnitude |Z| and phase of impedance response in the frequency range from 40 Hz to 1 MHz of impedance circuits consisting of reconfigurable YMO-based resistive switches and not reconfigurable passive elements: a commercial capacitor (C=0.047μF) in (**a**) parallel and (**b**) series configurations and commercial inductor (L=1000μH) in (**c**) parallel and (**d**) series configurations. In the upper and lower rows of each subfigure, the magnitude and phase of impedance circuits with YMO resistive switch in LRS and HRS, respectively, are shown.

Exploiting the reconfigurable resistive behavior of the YMO resistive switch, we have achieved distinctive impedance responses with tunable cut-off frequency features between LRS and HRS within the frequency domain, employing the same circuit topology. Figure 4 illustrates the magnitude and phase of impedance responses for the impedance circuit (depicted by black lines), featuring a single YMO resistive switch coupled with an external commercial electrical component, either a capacitor (C=0.047μF) or an inductor (L=1000μH). The magnitude and phase of impedance responses are presented in the left and right columns of each subfigure, respectively.

In each subfigure, we present the impedance responses of a single YMO resistive switch in its corresponding resistive state (green lines) and the impedance response of the individual electrical component (blue lines) as reference cases. Notably, in the impedance responses of a single YMO resistive switch (green lines), a pronounced difference in impedance magnitude is evident between the LRS and HRS states. Nevertheless, the phase of the single YMO resistive switch remains consistently at 0°, underscoring the prevailing resistive behavior in the YMO resistive switch regardless of its state (LRS or HRS). The rationale for selecting the aforementioned values for the two commercial capacitor or inductor components is that the transition frequency of the impedance circuitry can be experimentally determined within the testing frequency range spanning from 40 Hz to 1 MHz.

The magnitude and phase of impedance responses in the impedance circuitry featuring a YMO resistive switch connected in parallel (YMO//C) and in series (YMO+C) with a capacitor (C=0.047μF) is illustrated in Figure 4a and Figure 4b, respectively. In Figure 4a, distinct impedance responses are observed within the YMO//C circuitry, highlighting the transition between LRS (upper case) and HRS (lower case) states of the YMO resistive switch. The impedance behavior of the YMO//C circuitry shifts from resistive to the capacitive mode when the YMO resistive switch is in either LRS or HRS. Notably, there are significant differences in cut-off frequencies observed when the YMO resistive switch is in LRS or HRS, with corresponding transition frequencies experimentally recorded as $f_{t,LRS}=$ 5.6 × 10^4^ Hz and $f_{t,HRS}=$ 7.6 × 10^2^ Hz, respectively. Utilizing the parameters derived from the equivalent model in Figure 3d, the cut-off frequencies for the YMO//C circuit incorporating a YMO resistive switch, coupled with a capacitor (C=0.047μF), can be estimated using equations $f_{t}=\frac{1}{2\pi RC}$, respectively. The experimental results align well with the predictions from the equivalent circuit model. Similarly, Figure 4b demonstrates distinguishable impedance responses in the YMO+C circuitry when the YMO resistive switch is in LRS (upper case) or HRS (lower case). The transition points for both YMO//C and YMO+C configurations in LRS and HRS are experimentally identified at $f_{t,LRS}=$ 5.6 × 10^4^ Hz and $f_{t,HRS}=$ 7.6 × 10^2^ Hz, respectively. Utilizing the equation $f_{t}=\frac{1}{2\pi RC}$ with the modeled resistive value of YMO in LRS and HRS ($R_{LRS}=$ 40 Ω, $R_{HRS}=$ 5.2 × 10^3^ Ω) with the capacitance of the external capacitor (C=0.047μF), the computed $f_{t,LRS}$ and $f_{t,HRS}$ amount to 8.4 × 10^4^ Hz and 6.7 × 10^2^ Hz, respectively. We can conclude that the computed transient frequency in both states is well comparable with the experimentally recorded values. The difference in transient frequency between computed and experimental values possibly results from neglecting the parallel capacitors and resistors. The impedance behavior of the YMO+C circuitry undergoes a transition from the capacitive to resistive mode with the YMO resistive switch in both LRS and HRS. The experimentally recorded cut-off frequency for the YMO+C circuit is comparable to that of the YMO//C circuit, showing consistency between experimental tests and equivalent circuit modeling.

When utilizing an inductor (L=1000μH) as the external device, clear and distinguishable impedance responses are observed for the YMO//L and YMO+L configurations, illustrated in Figure 4c and Figure 4d, respectively. In the YMO//L configuration (Figure 4c), the larger inductance value of the external inductor (L=1000μH) results in an impedance phase that initially mirrors the response of a single commercial inductor in parallel within the low-frequency domain. Subsequently, it transitions to a resistive mode in the higher-frequency domain. In contrast, in the YMO+L configuration (Figure 4d), the impedance response follows that of a single YMO resistive switch and then transitions to an inductive mode. The transition points for both YMO//L and YMO+L configurations in LRS and HRS are experimentally identified at $f_{t,LRS}=$ 6.1 × 10^3^ Hz and $f_{t,HRS}=$ 2.3 × 10^5^ Hz, respectively. Utilizing the equation $f_{t}=\frac{R}{2\pi L}$ with the modeled resistive value of YMO in LRS and HRS ($R_{LRS}=$ 40 Ω, $R_{HRS}=$ 5.2 × 10^3^ Ω) with the inductance of the external inductor (L=1000μH), the computed $f_{t,LRS}$ and $f_{t,HRS}$ amount to 6.4 × 10^3^ Hz and 7.9 × 10^5^ Hz, respectively. We can conclude that the computed transient frequency in both states is comparable to the experimentally recorded values. The difference in transient frequency between computed and experimental values might result from the additional capacitive components visible in the modeling of YMO resistive switches in both states but not considered in the computation of transient frequency.

**Figure 5:**
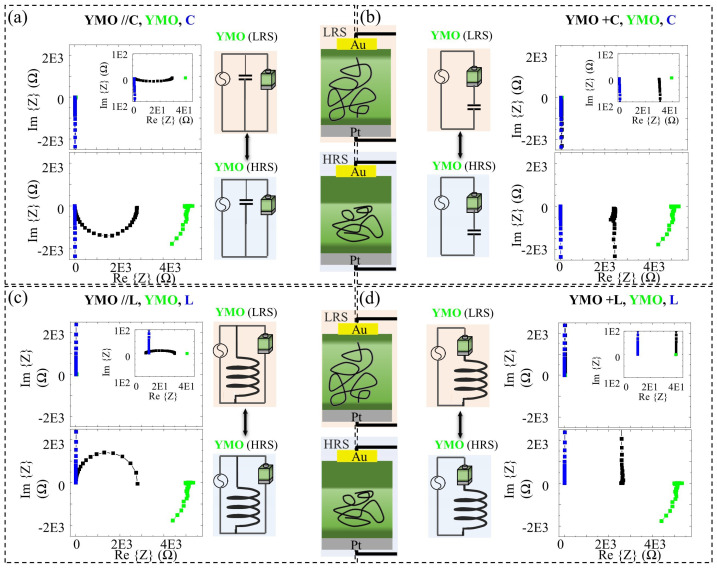
Measured Nyquist plots (Re{Z} vs. Im{Z}) in the frequency range from 40 Hz to 1 MHz of impedance circuits consisting of reconfigurable YMO-based resistive switches and not reconfigurable passive elements: a commercial capacitor (C=0.047μF) in (**a**) parallel and (**b**) series configurations and commercial inductor (L=1000μH) in (**c**) parallel and (**d**) series configurations. In the upper and lower rows of each subfigure, the magnitude and phase of impedance circuits with YMO resistive switch in LRS and HRS, respectively, are shown.

In Figure 5, we present experimental Nyquist plots illustrating the impedance characteristics of circuits incorporating YMO and commercial components such as capacitors (C=0.047μF) or inductors (L=1000μH) across a frequency range spanning from 40 Hz to 1 MHz. These plots offer valuable insights into the intricate dynamics between the real and imaginary components of the impedance response. Through these Nyquist plots, we gain a comprehensive understanding of the unique impedance responses linked to the reconfiguration of the resistive switch in the YMO-based circuits. Remarkably, the experimental recordings reveal substantial disparities in both the real and imaginary parts of the Nyquist plots when the YMO resistive switch transitions between LRS and HRS. These differences align seamlessly with the discernible shifts in impedance behavior, as demonstrated in Figure 4. This alignment serves as compelling evidence, highlighting the tunable frequency response exhibited by the YMO resistive switch-based impedance circuits in LRS and HRS.

Figure 5a,b presents Nyquist plots illustrating the impedance characteristics of a reconfigurable circuit incorporating a commercial capacitor (C=0.047μF) in either parallel (YMO//C) or series (YMO+C) with the YMO resistive switch, corresponding to the switch being in low-resistance states (LRS) or high-resistance states (HRS), respectively. Each subfigure includes Nyquist plots for the complete impedance circuits (in black), a single YMO resistive switch in LRS or HRS (in green), and a standalone commercial capacitor (C=0.047μF) (in blue). In Figure 5a, the Nyquist plots within the YMO//C circuitry reveal distinctive semicircles, indicating the transition between LRS (upper case) and HRS (lower case) of the YMO resistive switch. These Nyquist plots exhibit a semicircular pattern within the negative imaginary scale when the YMO resistive switch is in either LRS or HRS. Similarly, Figure 5b demonstrates distinguishable Nyquist plots within the YMO+C circuitry when the YMO resistive switch is in LRS (upper case) or HRS (lower case). The Nyquist plots of the YMO+C circuitry exhibit a straight line into the negative quadrant of the imaginary part of the impedance response, resembling the behavior of a standalone commercial capacitor. Notably, regardless of whether it is in the YMO//C or YMO+C circuitry, the real and imaginary parts of the Nyquist plots are too small to be visible in LRS (upper cases) compared to the values recorded in HRS (lower cases). Consequently, a zoomed-in inset in Figure 5a,b is provided to visualize the semicircular pattern.

Figure 5c,d depicts Nyquist plots representing the impedance characteristics of an impedance circuit incorporating a commercial inductor (L=1000μH) in either parallel (YMO//L) or series (YMO+L) with the YMO resistive switch, corresponding to the switch being in LRS or HRS, respectively. In Figure 5c, the Nyquist plots within the YMO+L circuitry showcase distinct semicircles within the positive imaginary scale, indicating the transition between LRS (upper case) and HRS (lower case) of the YMO resistive switch. The Nyquist plots of the YMO+L circuitry exhibit a semicircular pattern when the YMO resistive switch is in either LRS or HRS. In Figure 5d, evident disparities are observed in the Nyquist plots within the YMO+L circuitry contingent on whether the YMO resistive switch is in a low-resistance state (LRS, upper case) or a high-resistance state (HRS, lower case). The Nyquist plots for the YMO+L circuitry exhibit a linear pattern within the positive quadrant of the imaginary part of the impedance response, closely resembling the characteristic behavior of an independent commercial inductor. In both YMO//L and YMO+L circuit configurations, the real and imaginary components of the Nyquist plots are notably minimal during LRS (upper cases), making them imperceptible in comparison to the values recorded during HRS (lower cases). This disparity in values necessitates the incorporation of a zoomed-in inset in Figure 5c,d to facilitate the visualization of the linear response in the positive imaginary quadrant.

The exhibited distinctive impedance responses of the impedance circuits utilizing the YMO resistive switch underscore its remarkable potential for tunable frequency responses. This reconfigurability, inherent in the YMO resistive switch, paves the way for the development of versatile impedance circuits. These reconfigurable circuits hold significant promise for diverse electronic applications, particularly in the domain of filter circuitry, where the tunability of frequency responses is a crucial attribute. The demonstrated capabilities of the YMO resistive switch-based impedance circuits open up avenues for innovative designs in electronic systems, offering enhanced flexibility and adaptability for tailored frequency-dependent functionalities.

## 4. Conclusions

In this investigation, we thoroughly explored the distinctive characteristics of electroforming-free YMO-based resistive switches, highlighting their capacity for reconfigurable memory effects and, notably, their pivotal role in achieving tunable frequency responses within impedance circuits. We measured and modeled the impedance of YMO-based resistive switches wire-bonded to a diode socket and showed that contact resistance is decreased by more than 100 times when switching the YMO-based resistive switch from HRS to LRS. We anticipate that the resistive switching from HRS to LRS is related to a restructuring of the network of vortices in YMO until the first vortex shunts the top and bottom electrode of the YMO-based resistive switch. Moving beyond the scope of individual resistive switches, we seamlessly integrated YMO resistive switches with commercially available capacitors and inductors, crafting reconfigurable M–C and M–L circuits, respectively. Our in-depth analysis of impedance responses elucidated the tunable frequency response exhibited by these circuits when the YMO resistive switch operates in either the LRS or HRS. The visual representation of Nyquist plots provided a clear illustration of the intricate dynamics between real and imaginary components, offering invaluable insights into the reconfigurable behavior inherent in these circuits.

The demonstrated adaptability in frequency responses, made feasible by the distinctive properties of YMO resistive switches, not only highlights their immediate potential but also lays the groundwork for pioneering designs in electronic systems. This capability propels the field towards the development of more advanced and flexible electronic technologies, promising substantial progress in the domain of electronic circuits and devices. As a result, the versatility of YMO resistive switches in achieving tunable frequency responses introduces a paradigm shift in electronic design, providing researchers and engineers with a powerful tool to tailor circuit functionalities for specific application requirements.

## Data Availability

Data are available upon request.
